# Emerging insights into inflammatory bowel disease from the intestinal microbiota perspective: a bibliometric analysis

**DOI:** 10.3389/fimmu.2023.1264705

**Published:** 2023-10-26

**Authors:** Anqi Zhang, Fang Wang, Delong Li, Chong-Zhi Wang, Haiqiang Yao, Jin-Yi Wan, Chun-Su Yuan

**Affiliations:** ^1^ School of Traditional Chinese Medicine, Beijing University of Chinese Medicine, Beijing, China; ^2^ National Institute of Traditional Chinese Medicine (TCM) Constitution and Preventive Medicine, Beijing University of Chinese Medicine, Beijing, China; ^3^ Department of Traditional Chinese Medicine, 731 Hospital of China Aerospace Science and Industry Group, Beijing, China; ^4^ Tang Center for Herbal Medicine Research, The University of Chicago, Chicago, IL, United States; ^5^ Department of Anesthesia and Critical Care, The University of Chicago, Chicago, IL, United States

**Keywords:** intestinal microbiota, inflammatory bowel disease (IBD), bibliometric analysis, citation, research trends

## Abstract

**Background:**

Inflammatory bowel disease (IBD) has caused severe health concerns worldwide. Studies on gut microbiota have provided new targets for preventing and treating IBD. Therefore, it is essential to have a comprehensive understanding of the current status and evolution of gut microbiota and IBD studies.

**Methods:**

A bibliometric analysis was performed on documents during 2003-2022 retrieved from the Scopus database, including bibliographical profiles, citation patterns, and collaboration details. Software programs of VOSviewer, CiteSpace, and the Bibliometrix R package visually displayed the mass data presented in the scientific landscapes and networks.

**Results:**

10479 publications were retrieved, showing a steadily growing tendency in interest. Xavier Ramnik J. group led the total number of publications (73 papers) and 19787 citations, whose productive work aroused widespread concern. Among the 1977 academic journals, the most prolific ones were *Inflammatory Bowel Diseases*, *Frontiers in Immunology*, and *Nutrients*. Research outputs from the United States (US, 9196 publications), China (5587), and Italy (2305) were highly ranked.

**Conclusion:**

Our bibliometric study revealed that the role of gut microbiota has become a hot topic of IBD research worldwide. These findings are expected to improve understanding of research characteristics and to guide future directions in this field.

## Introduction

Inflammatory bowel disease (IBD) comprises a heterogeneous group of inflammatory disorders that are immune-mediated and primarily affect the gastrointestinal tract, with Crohn’s disease (CD) and ulcerative colitis (UC) being the two main subtypes (1, 2). IBD significantly impacts daily life and is a significant risk factor for the development of gastrointestinal cancers (3). The rapid evolution of social norms, lifestyles, diets, and the environment resulting from contemporary human behavior may instigate or contribute to the escalating prevalence of IBD, rendering it an emerging global concern (4). Increasing evidence suggests that the gut microbiome plays a crucial role in the development of IBD (5). IBD is associated with alterations in the gut microbiome, characterized by a consistent reduction in bacterial diversity (6). Meanwhile, fecal microbiota transplantation has been shown to restore intestinal microecological balance and treat IBD effectively (7).

Microbiota in the human digestive tract make up a complex ecological system. To date, over 3000 species have been detected in human feces; only 30% of this bacterial population is the typical core microflora shared between different individuals (8, 9). Investigations have indicated that gut microbiota is crucial in the maintenance of intestinal physiological function (10). The dynamic composition of the microbiota is influenced and regulated by a combination of endogenous and exogenous factors (11). Diet, hormones, medication, and health conditions of the host may affect the numbers and diversity of microflora in the gastrointestinal tract (10, 11). Dramatic perturbations like these may result in dysbiosis characterized by an altered composition and reduced stability (12). Moreover, microbiota dysbiosis could induce various human diseases like IBD in the pathological processes (13, 14).

Bibliometric analysis is an approach to evaluate the trends and characteristics of published literature in a particular domain over time. It provides an easy and direct way for scientists and researchers to access the field’s developing trends and research interests. The academic influence of leading publications and literature distributions from different origins is clearly present (15, 16). The conventional classification and summarization of literature heavily rely on the subjective judgment of authors, making it challenging to analyze a large volume of literature comprehensively and accurately. To address this issue, scientific cartography based on bibliometric quantitative analysis can be employed to examine the structure and development of research fields. This method facilitates the summarization and analysis of applied literature while uncovering key application areas and enables topic clustering using CiteSpace or VOSviewer software (17). Currently, bibliometric analysis has garnered increasing attention due to its distinctive advantages that enable investigators to delve into specific fields of study through the visualized analysis of citations, co-citations, geographic distribution, and term frequency, yielding highly valuable insights (18).

In this study, on the hotspot of gut microbiota and IBD, we conducted a bibliometric analysis of publications in the Scopus database during the past two decades to capture its research state and trends. Using software programs VOSviewer, CiteSpace, and Bibliometrix, we mapped the literature landscape and distribution layouts of active authors, journals, institutions, and countries. We also visualized the patterns of cooperation and citation. This study presents an overview and summary of the evolution of gut microbiota and IBD studies, and analyzes the current research state and future trends, aiming to assist researchers and policymakers gain a comprehensive understanding of the study on this topic and better grasp future directions.

## Methods

### Data source

A bibliometric search of research output on the gut microbiome and IBD, published from 2003 to 2022, was performed on July 10, 2023, using the Scopus database. Scopus by Elsevier is known to be the most comprehensive data source for detailed bibliometric evaluation from a quantitative and qualitative point of view (19–21). With the comprehensive coverage of scientific journals and the powerful performance of analytical tools, Scopus was selected as the literature source to retrieve abstracts, citations, and other bibliometric data at the initial stage.

### Search strategy

Aiming to ensure reliable and accurate records, our primary keywords used in the literature search focused on gut microbiota and inflammation, along with the relevant synonyms based on Medical Subject Headings (MeSH) in MEDLINE (22). The terms “gastrointestinal microbiomes,” “gut microflora,” “gut microbiota,” “gastrointestinal flora,” “gut flora,” “gastrointestinal microbiota,” “gut microbiome,” “gastrointestinal microflora,” “intestinal microbiome,” “intestinal microbiota,” “intestinal microflora,” “intestinal flora” and “enteric bacteria” were used as the keywords of gut microbiota; the primary keywords of IBD were “inflammatory bowel disease” and “IBD” Meanwhile also includes “ulcer colitis”, “UC”, “Crohn disease” and “CD”. The two sets of keywords with the AND logic were searched in the field of “Article title/Abstract/Keywords.” The search was conducted in Scopus using the following terms: (TITLE-ABS-KEY (gastrointestinal AND microbiomes) OR TITLE-ABS-KEY (gut AND microflora) OR TITLE-ABS-KEY (gut AND microbiota) OR TITLE-ABS-KEY (gastrointestinal AND flora) OR TITLE-ABS-KEY (gut AND flora) OR TITLE-ABS-KEY (gastrointestinal AND microbiota) OR TITLE-ABS-KEY (gut AND microbiome) OR TITLE-ABS-KEY (gastrointestinal AND microflora) OR TITLE-ABS-KEY (intestinal AND microbiome) OR TITLE-ABS-KEY (intestinal AND microbiota) OR TITLE-ABS-KEY (intestinal AND microflora) OR TITLE-ABS-KEY (intestinal AND flora) OR TITLE-ABS-KEY (enteric AND bacteria) AND PUBYEAR > 2002 AND PUBYEAR < 2023) AND (TITLE-ABS-KEY (inflammatory bowel disease) OR TITLE-ABS-KEY (ulcer colitis) OR TITLE-ABS-KEY (crohn disease) OR TITLE-ABS-KEY (IBD) OR TITLE-ABS-KEY (UC) OR TITLE-ABS-KEY (CD) AND PUBYEAR > 2002 AND PUBYEAR < 2023).

### Data analysis

The search outcomes from the Scopus database were exported into CSV format for further analysis, including bibliographical profiles, citation patterns, collaboration details, and other retrieved publications. We established the inclusion criteria as follows: 1. The literature pertains to topics of IBD and inflammation; 2. Articles published within the past two decades (2003-2022). Exclusions encompassed: 1. Incomplete or duplicated literature; 2. Non-academic documents such as conference proceedings, calls for papers, news reports, patent achievements, and newspaper abstracts.

Microsoft Excel and GraphPad Prism (Version 9.5.0, San Diego, CA, US) were applied to conduct statistical procedures, generating frequency distribution, sum, and average data. Further investigations were performed to determine the top, most prolific authors, journals, countries, institutions, and the most cited papers according to the standard competition ranking (SCR, also known as the 1-2-2-4 rule). We calculated the H-index to assess the number and level of academic output of researchers. In addition, we also calculate the G index and M-index as a supplement to the H index. The calculation method of G-index is as follows: the papers are sorted in descending order according to the number of citations, and the number of citations is superimposed according to the serial number. When the cumulative number of citations is equal to the square of the serial number, the serial number value is the G-index (23). The M-index is derived from the H-index of academic tenure, calculated by dividing the H-index by the number of years since an author’s initial publication (24).

**Figure 1:**
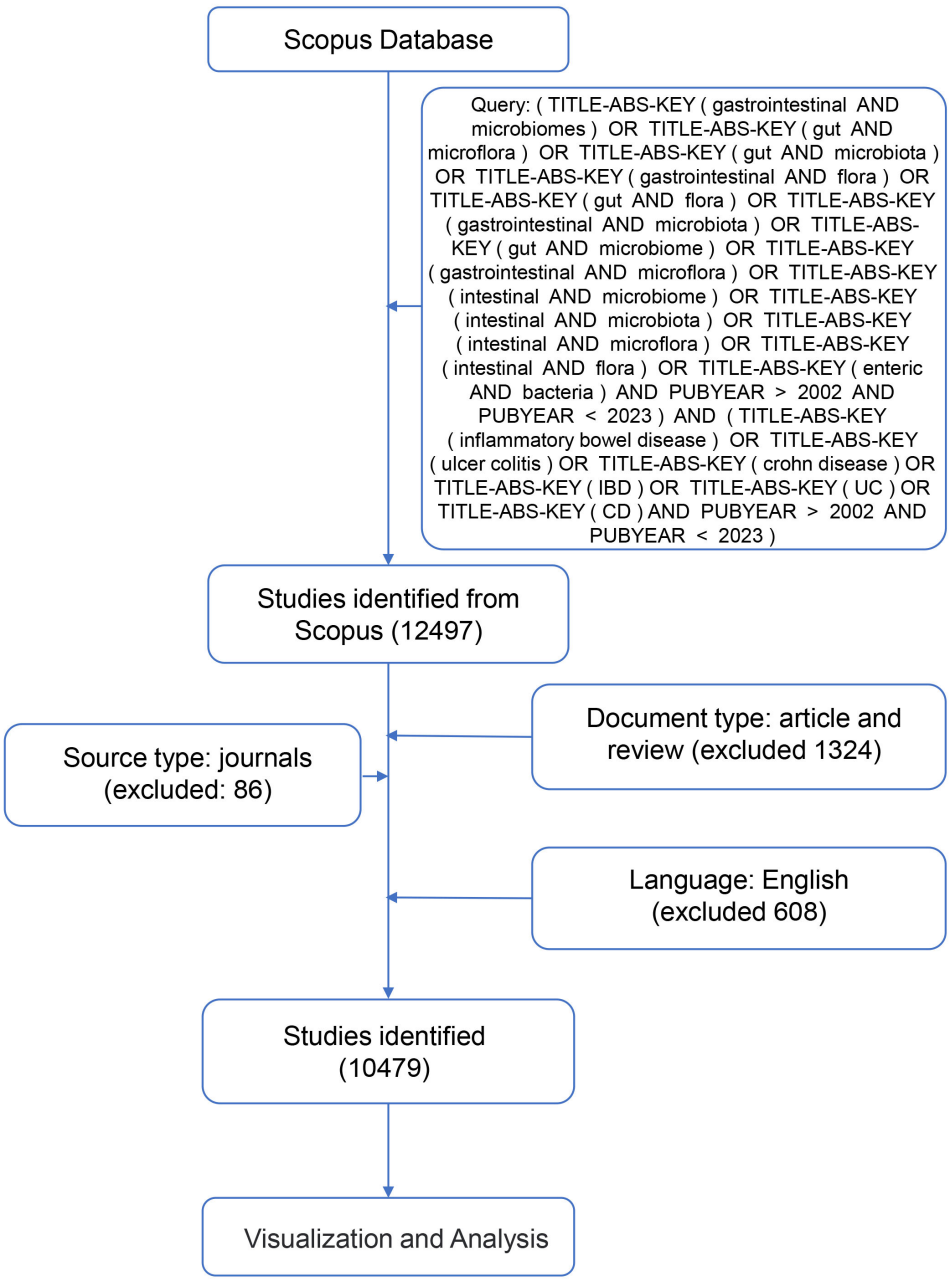
Flow diagram of the literature selection process in this study.

Visualization analysis was applied for presenting a mass of data to display scientific landscapes and networks using software programs of VOSviewer (v.1.6.17) (25), CiteSpace (v.5.8.R2) (26), and the R package of Bibliometrix (27). VOSviewer conducts a visual analysis of country, institutional, author, and collaboration distribution, as well as keyword collaboration networks. Clustering is automatically completed using the similarity matrix and mapping techniques of VOSviewer, with corresponding labels added by the authors based on content. CiteSpace was utilized to analyze the distribution and collaboration among countries, institutions, keyword timelines, and reference data. Additionally, we employ R studio Desktop Software (v.2023.6.1.0) linked to the R Software (v.4.3.1) and converted into an R data frame. The Bibliometrix R package, which provides a web interface, was used for statistical analysis of the number of publications, references, and other data and visual analysis of national distribution and cooperation (27). The flow diagram for the searching and sorting process of related articles is shown in **Figure 1**. All raw data utilized in this study were sourced from publicly available databases, thus exempting the need for ethical review.

## Results

### Overview and trends in research literature production

**Figure 2:**
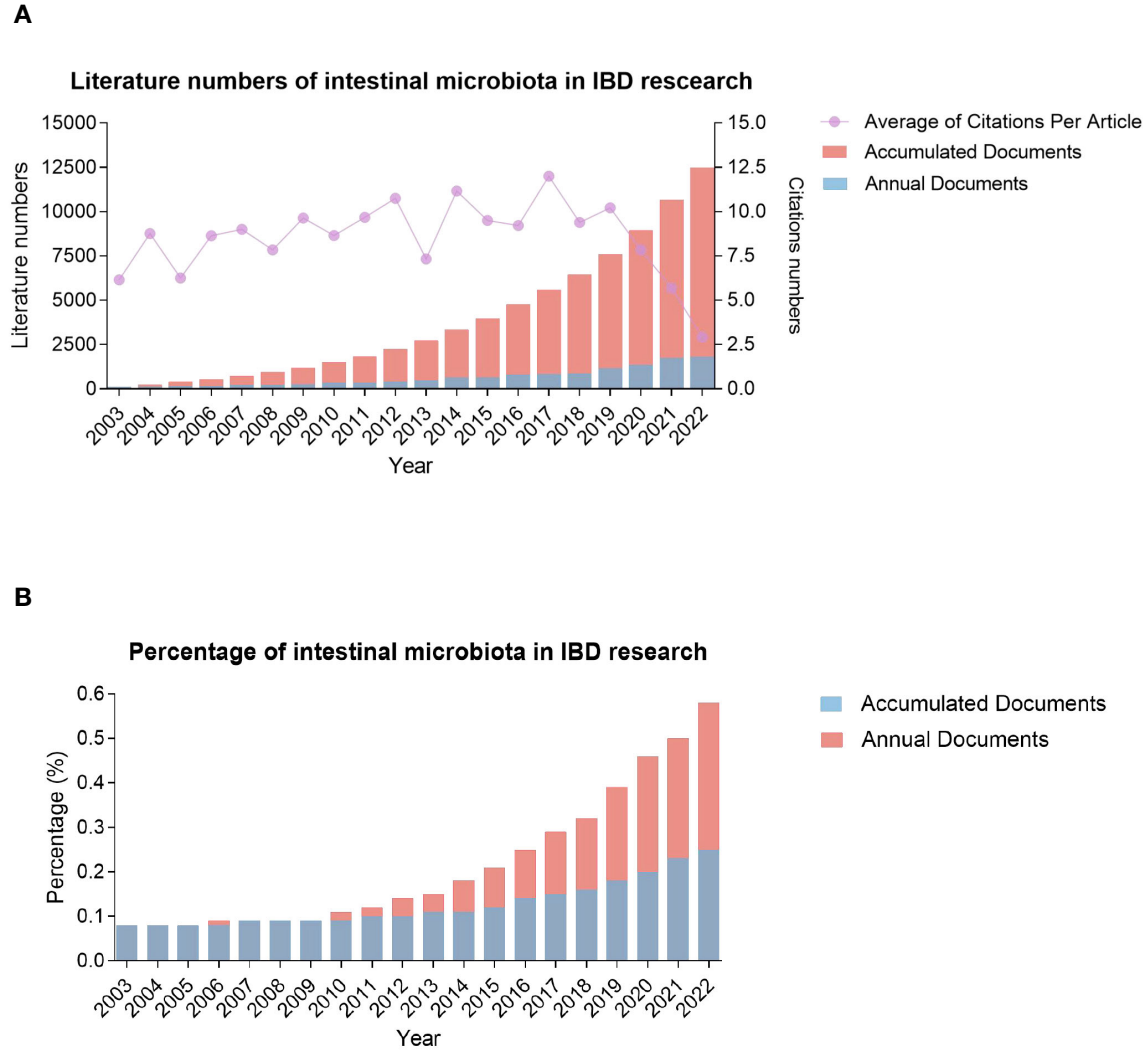
Global trends in the number of published articles related to gut microbiota and IBD over the past two decades from 2003 through 2022. **(A)** annual and accumulated publications of intestinal microbiota and IBD; **(B)** the percentage of intestinal microbiota-related publications in the IBD research.

The 20-year period 2003-2022 saw the publication of 10479 articles in gut microbiota and IBD research. Global trends in the number of annual and accumulated publications related to this topic are shown in **Figure 2A**. During the first four years, 2003-2006, the number of annual articles ranged from117 to 156 and was relatively stable, implying that this crossover domain was not so attractive to scientists at that moment. Moreover, within a span of only two years (2007-2008), two important programs of the Human Microbiome Project (HMP) were launched by the United States National Institutes of Health (NIH) in 2007 (28), and Metagenomics of the Human Intestinal Tract (MetaHIT) by European Union in 2008 (29), and the number of annual articles increased to 209. Since then, the number has continued to rise, reaching 1813 publications in 2022 (or 906.5% of the articles in 2008). The active interest and intensive efforts from worldwide research communities in the past ten years have led to the enormous growth of this field, which is supported by a significant increase in the number of related publications. The number of articles published on this topic in the recent decade is more than 5.93 times in the first ten years since 2003. Furthermore, it is noted that the percentage of annual intestinal microbiota-related publications in the domain of inflammation research was increasing gradually, starting from 2009, when an increase from 0.9%-0.11% was observed (**Figure 2B**). Therefore, the combination of gut microbiota and inflammation as an immediate area of the research focus has attracted significant attention from scientists worldwide.

**Figure 3:**
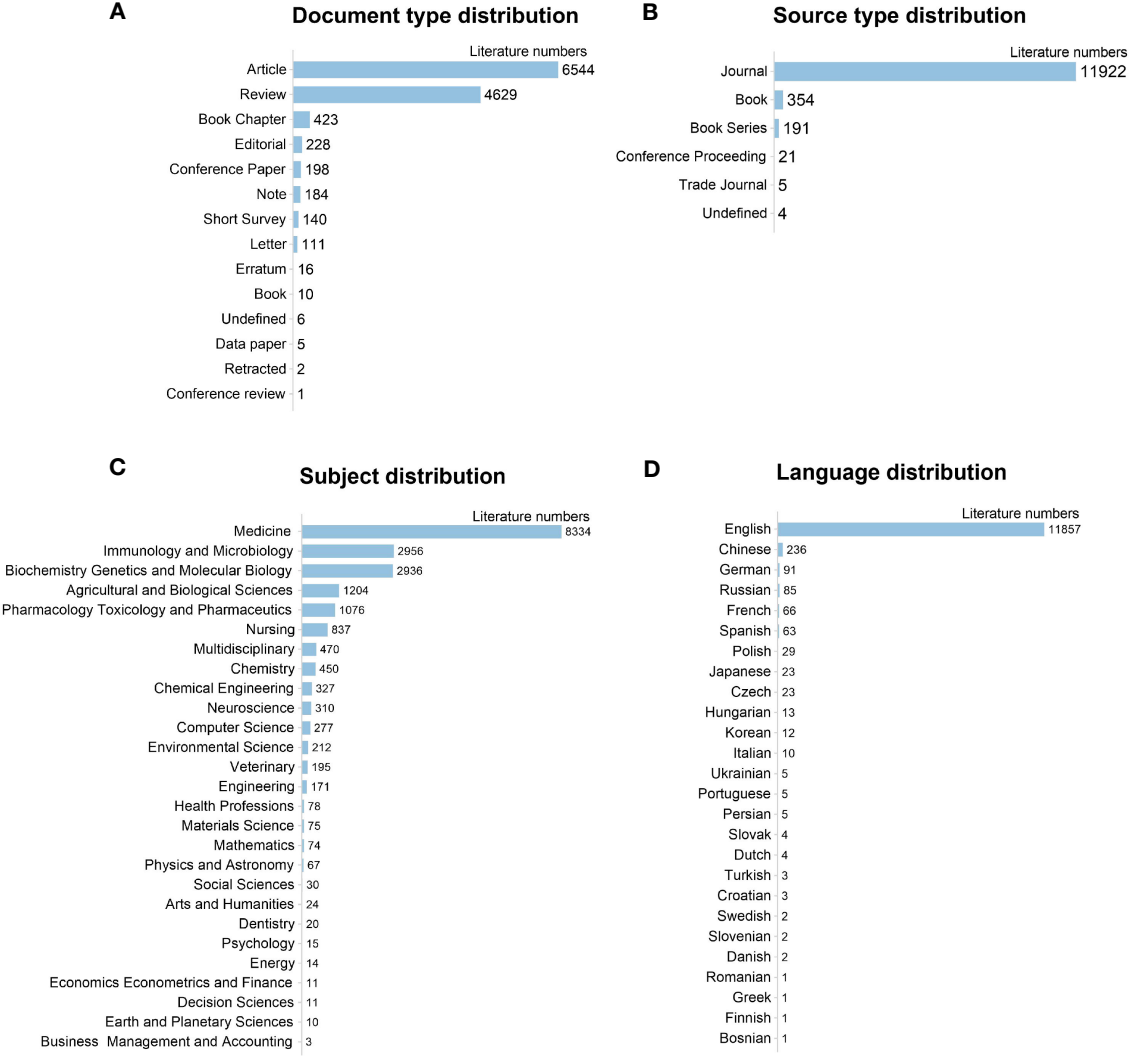
General information of retrieved 10479 publications on gut microbiota and IBD. **(A)** Document type distribution; **(B)** Source type distribution; **(C)** Subject distribution; **(D)** Language distribution.

Among the retrieved documents, the research articles (6544, 52.36%) and reviews (4629, 37.04%) made up the majority, while the others (10.60%) were conference papers, book chapters, short surveys, notes, and editorials, etc. (**Figure 3A**). Journals were the primary source of documents, accounting for 95.40% of all the publications (**Figure 3B**). In terms of subject distribution (**Figure 3C**), 8334 documents (66.69%) were related to Medicine, 2936 (23,49%) to Biochemistry, Genetics, and Molecular Biology, 2956 (23.65%) to Immunology and Microbiology, and 1204 (9.63%) to Agricultural and Biological Sciences. Of the 26 languages published, English was predominant (11857, 94.88%), followed by Chinese (236, 1.89%). Other languages, like German, Russian, French, etc., only covered less than 1% of publications (**Figure 3D**).

Due to the source type and language heterogeneity among the retrieved documents, we set the inclusion criteria to limit publications to only research articles written in English to perform further analyses. Thus, papers of document types besides research articles and papers written in other languages were excluded (**Figure 1**). As a result, a total of 10479 English papers were included for the following analyses.

### Analysis of the most cited articles

Although many factors may influence the citation impact of publications, it is widely regarded as a vital evaluation index for scientific documents. **Table 1** presents the 20 most commonly cited papers between 2003 and 2022 (30–49). “Metabolic endotoxemia initiates obesity and insulin resistance,” published in *Nature* by *David L.A. et al.*, was the most frequently cited article (5975 times) (30). In addition, the top journals were represented among the most cited articles in this field. Of the 20 most cited papers, four were published in *Nature*, and three were published in *Proceedings of the National Academy of Sciences of the United States of America* (*PNAS*), two from *NEJM* and two from *Cell*, respectively.

**Table 1:** Top 20 most cited articles on gut microbiota and IBD from 2003 to 2022.

SCR	Authors	Title	Year	Journals	Citations
1^st^	David et al.	Diet rapidly and reproducibly alters the human gut microbiome	2014	*Nature*	5975
2^nd^	Kaper et al.	Pathogenic Escherichia coli	2004	*Nat Rev Microbiol*	3500
3^rd^	Round et al.	The gut microbiota shapes intestinal immune responses during health and disease	2009	*Nat Rev Immunol*	3402
4^th^	Frank et al.	Molecular-phylogenetic characterization of microbial community imbalances in human inflammatory bowel diseases	2007	*PNAS*	3341
5^th^	Lozupone et al.	Diversity, stability and resilience of the human gut microbiota	2012	*Nature*	3247
6^th^	Sokol et al.	Faecalibacterium prausnitzii is an anti-inflammatory commensal bacterium identified by gut microbiota analysis of Crohn disease patients	2008	*PNAS*	3034
7^th^	Guarner et al.	Gut flora in health and disease	2003	*Lancet*	2528
8^th^	Clemente et al.	The impact of the gut microbiota on human health: An integrative view	2012	*Cell*	2405
9^th^	Cho et al.	The human microbiome: At the interface of health and disease	2012	*Nat Rev Genet*	2185
10^th^	Abraham et al.	Inflammatory bowel disease	2009	*NEJM*	2184
11^st^	Gevers et al.	The treatment-naive microbiome in new-onset Crohn’s disease	2014	*Cell Host and Microbe*	2025
12^nd^	O’Hara et al.	The gut flora as a forgotten organ	2006	*EMBO Reports*	1948
13^rd^	Lynch et al.	The human intestinal microbiome in health and disease	2016	*NEJM*	1861
14^th^	Morgan et al.	Dysfunction of the intestinal microbiome in inflammatory bowel disease and treatment	2012	*Genome biology*	1828
15^th^	Khor et al.	Genetics and pathogenesis of inflammatory bowel disease	2011	*Nature*	1765
16^th^	Mazmanian et al.	A microbial symbiosis factor prevents intestinal inflammatory disease	2008	*Nature*	1746
17^th^	Manichanh et al.	Reduced diversity of fecal microbiota in Crohn’s disease revealed by a metagenomic approach	2006	*Gut*	1683
18^th^	Round et al.	Inducible Foxp3+ regulatory T-cell development by a commensal bacterium of the intestinal microbiota	2010	*PNAS*	1617
19^th^	Roberfroid et al.	Prebiotic effects: Metabolic and health benefits	2010	*British Journal of Nutrition*	1510
20^th^	Elinav et al.	NLRP6 inflammasome regulates colonic microbial ecology and risk for colitis	2011	*Cell*	1493

SCR, standard competition ranking; PNAS, Proceedings of the National Academy of Sciences of the United States of America; NEJM, New England Journal of Medicine; Nat Rev Microbiol, Nature Reviews Microbiology; Nat Rev Immunol, Nature Reviews Immunology; Nat Rev Genet, Nature Reviews Genetics; IF, impact factor.

*Data extracted from Journal Citation Reports, Thomson Reuters, 2022.

### Contribution of author performance

**Figure 4:**
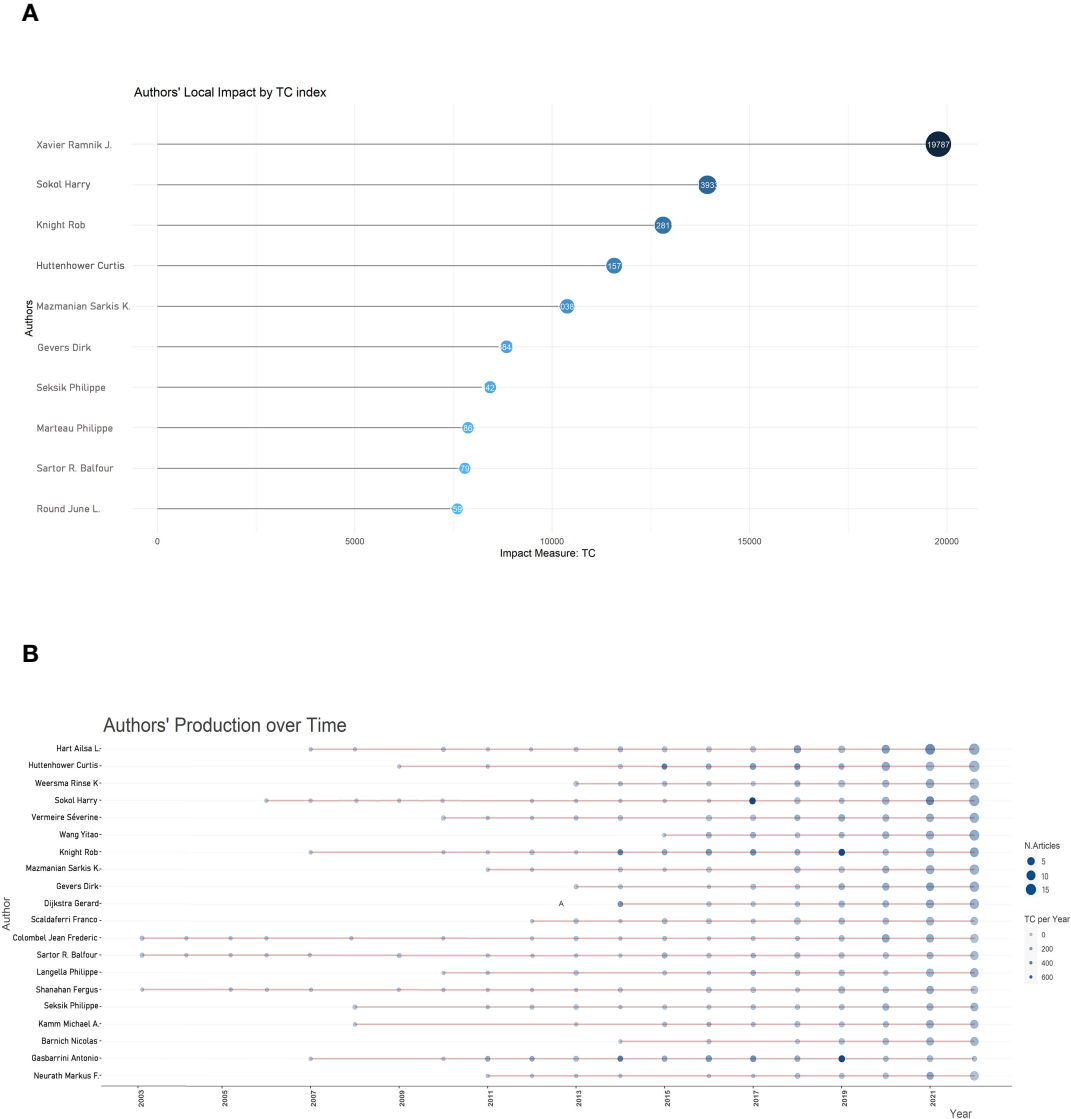
Bubble diagram depicting the influence of authors. **(A)** Top 10 authors with the highest citations; **(B)** Annual publication count and citation frequency. Larger shapes display more publications, and darker blue displays more citations.

A total of 36335 different authors contributed to the 10479 papers included in this study. Further analysis revealed that the 722 most prolific authors who published ten articles or more accounted for 2.0% of all contributors. We created a historical map of the related publications and authors in the format of a bubble chart. This chart demonstrated the 20 most productive authors by year, as shown in **Figure 4**. Since 2003, Sartor R. Balfour, Colombel Jean Frederick, and Shanahan Ferguson have been pioneers in exploring the field of gut microbiota and inflammation, but at that time there were relatively few related papers on this topic. After a 5-year significant increase from 2007 to 2012, this emerging field witnessed explosive publication growth from 2013. More specifically, Xavier Ramnik J. group led in the total number of publications (73 papers), followed by Sokol Harry (67), Sartor R. Balfour (50), Colombel Jean-Frederic (49), and Huttenhower Curtis (42).

The citation of the 234 most prolific authors with at least twenty publications was quantified and subjected to analysis, while the top 10 authors with the most published papers and the top 10 most cited authors on gut microbiota and IBD from 2003 to 2022 were presented in **Table 2**. Xavier, Ramnik J. led in the first place (19787 cited), followed by Sokol, Harry (13933) and Huttenhower, Curtis (11575). In addition, Xavier, Ramnik J. had the most citations (19787) among the top 20 most cited authors, followed by Sokol, Harry (13933) and Knight, Rob (12811). The 234 most prolific authors were also integrated into collaborative networks, as shown in **Figure S1**. The link thickness between any two authors indicates the extent of co-authorships (collaboration). The clusters in **Figure S1** revealed a strong correlation between the number of publications produced by an author and co-authorship. In other words, the more muscular the total link strength of scientific collaboration, the more authored publications.

**Table 2:** Top 10 prolific authors and top 10 most cited authors on gut microbiota and IBD from 2003 to 2022.

Rank	Author	H-index	G-index	M-index	TC	NP	PY start
1^st^	Xavier Ramnik J.	48	73	2.824	19787	73	2007
2^nd^	Sokol Harry	39	67	2.167	13933	67	2006
3^rd^	Sartor R. Balfour	33	50	1.571	7791	50	2003
4^th^	Colombel Jean Frederic	33	49	1.571	6200	49	2003
5^th^	Shanahan Fergus	32	43	1.524	7425	43	2003
6^th^	Huttenhower Curtis	35	42	2.692	11575	42	2011
7^th^	Vermeire Séverine	26	38	1.368	5395	38	2005
8^th^	Seksik Philippe	25	37	1.25	8427	37	2004
9^th^	Kamm Michael A.	28	37	1.333	5703	37	2003
10^th^	Neurath Markus F.	25	37	1.19	4972	37	2003
1^st^	Xavier Ramnik J.	48	73	2.824	19787	73	2007
2^nd^	Sokol Harry	39	67	2.167	13933	67	2006
3^rd^	Knight Rob	24	33	2	12811	33	2012
4^th^	Huttenhower Curtis	35	42	2.692	11575	42	2011
5^th^	Mazmanian Sarkis K.	12	13	0.706	10381	13	2007
6^th^	Gevers Dirk	19	20	1.462	8848	20	2011
7^th^	Seksik Philippe	25	37	1.25	8427	37	2004
8^th^	Marteau Philippe	19	22	0.95	7863	22	2004
9^th^	Sartor R. Balfour	33	50	1.571	7791	50	2003
10^th^	Round June L.	10	12	0.625	7599	12	2008

TC, total cited; NP, number of publications; PY start, publication year start.

*Data extracted from Journal Citation Reports, Thomson Reuters, 2022.

### Contribution of journal production

The retrieved articles were published in 1977 different academic journals. **Table 3** presents the top 20 active journals publishing articles on intestinal microflora and IBD, which produced 3269 articles (31.1%). Among them, *Inflammatory Bowel Diseases* took the leading position with 324 papers, followed by *Frontiers in Immunology* (301) and *Nutrients* (259). These three journals issued 8.5% of the total publications. According to IF, *Nature Reviews Gastroenterology and Hepatology* held the top position by an overwhelming high value of 65.1, owing to its excellent specialization in this field. In addition, Bradford’s law of scattering was applied here to reveal the distribution of the scientific literature in the research on gut microbiota and inflammation. Bradford zones acted as concentric zones of publication productivity with decreasing correlation, while each zone involved a similar number of articles. As shown in **Table 4** and **Figure S4**, a total of 1977 journals were distributed in 3 Bradford’s zones in the field of gut microbiota and IBD. The average number of articles in each zone was 3493.

**Table 3:** Top 20 prolific journals in publishing papers on intestinal microflora and IBD.

SCR	Journals	Documents	% N=10497	IF 2022
1^st^	*Inflammatory Bowel Diseases*	324	3.1%	4.9
2^nd^	*Frontiers in Immunology*	301	2.9%	7.3
3^rd^	*Nutrients*	259	2.5%	5.9
4^th^	*World Journal of Gastroenterology*	220	2.1%	4.3
5^th^	*Gut Microbes*	196	1.9%	12.2
6^th^	*Frontiers in Microbiology*	193	1.8%	5.2
7^th^	*Plos One*	190	1.8%	3.7
8^th^	*International Journal of Molecular Sciences*	179	1.7%	5.6
9^th^	*Gastroenterology*	139	1.3%	29.4
10^th^	*Gut*	128	1.2%	24.5
11^th^	*Journal of Crohn’S and Colitis*	100	1.0%	8.0
11^th^	*Scientific Reports*	100	1.0%	4.6
13^rd^	*Current Opinion in Gastroenterology*	98	0.9%	2.5
14^th^	*Frontiers in Cellular and Infection Microbiology*	92	0.9%	5.7
15^th^	*Mucosal Immunology*	85	0.8%	8.0
16^th^	*Digestive Diseases and Sciences*	77	0.7%	3.1
17^th^	*Food and Function*	76	0.7%	6.1
18^th^	*Frontiers in Pharmacology*	74	0.7%	5.6
19^th^	*Nature Reviews Gastroenterology and Hepatology*	73	0.7%	65.1
20^th^	*Cells*	67	0.6%	6.0

**Table 4:** Distribution of the journals in Bradford’s zones.

Bradford’s Zones	Number of Journals	% Journals	Number of articles	Bradford’s multiplier
1	29	1.4	3462	
2	199	10.1	3492	1.80
3	1749	88.5	3525	1.68
Total number of journals = 1977 Average number of articles in each zone = 3493				

**Figure 5:**
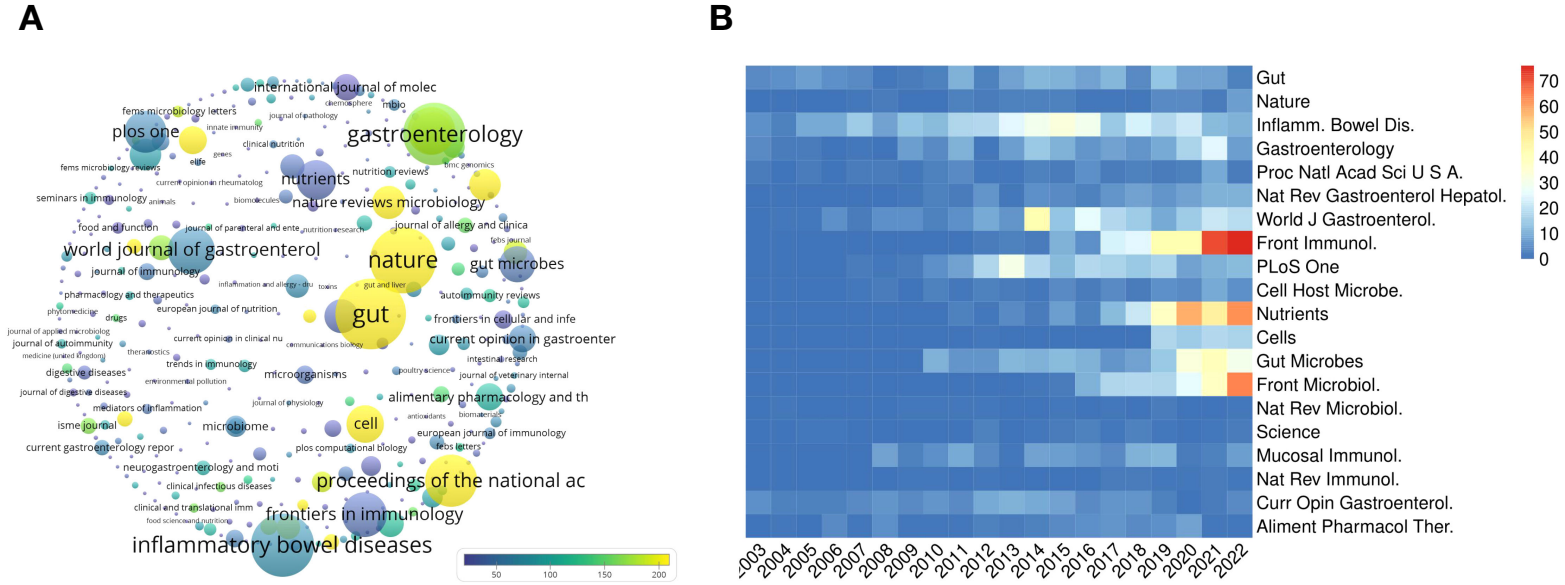
Citation analysis of journals. **(A)** Density map of 380 journals with a minimum productivity of 5 publications in this field. Journals with a higher number of citations have a yellower spot. **(B)** Heatmap illustrated the publication volume distributions per year of the top 20 most cited journals. The intensity of the blue hue is inversely proportional to the quantity of published papers, while the intensity of the red hue is directly proportional to the quantity of published papers.

The number of citations also indicates the power and authority of journals in the field. Citation analysis of 380 journals with minimum productivity of 5 publications was presented in **Figure 5A**. Articles on gut microbiota and IBD published in *Gut* received the highest number of citations (28430), while those published in *Nature* (25407) and *Inflammatory bowel disease* (23871) ranked second and third, respectively. The dot size is proportionate to the number of citations, with yellow indicating higher average citations and blue indicating lower average citations (**Figure 5A**).

Analyzing the top 20 most cited journals by year provided further insight into the level of journals directed to topic interest in **Figure 5B**. It was apparent that the journal citations have increased enormously, based on the great concentration in this area from 2008, which was consistent with patterns shown in **Figure 1**. Among the top 20 most cited journals, the quantity of publications regarding intestinal microflora and IBD has exhibited a consistent upward trend from 2003 to 2022. *Frontiers in Immunology, Nutrients*, and *Frontiers in Microbiology* experienced the most significant surge, demonstrating the most published research topics in intestinal microflora and IBD between 2003-2022.

### Global contribution and leading countries/regions

**Figure 6:**
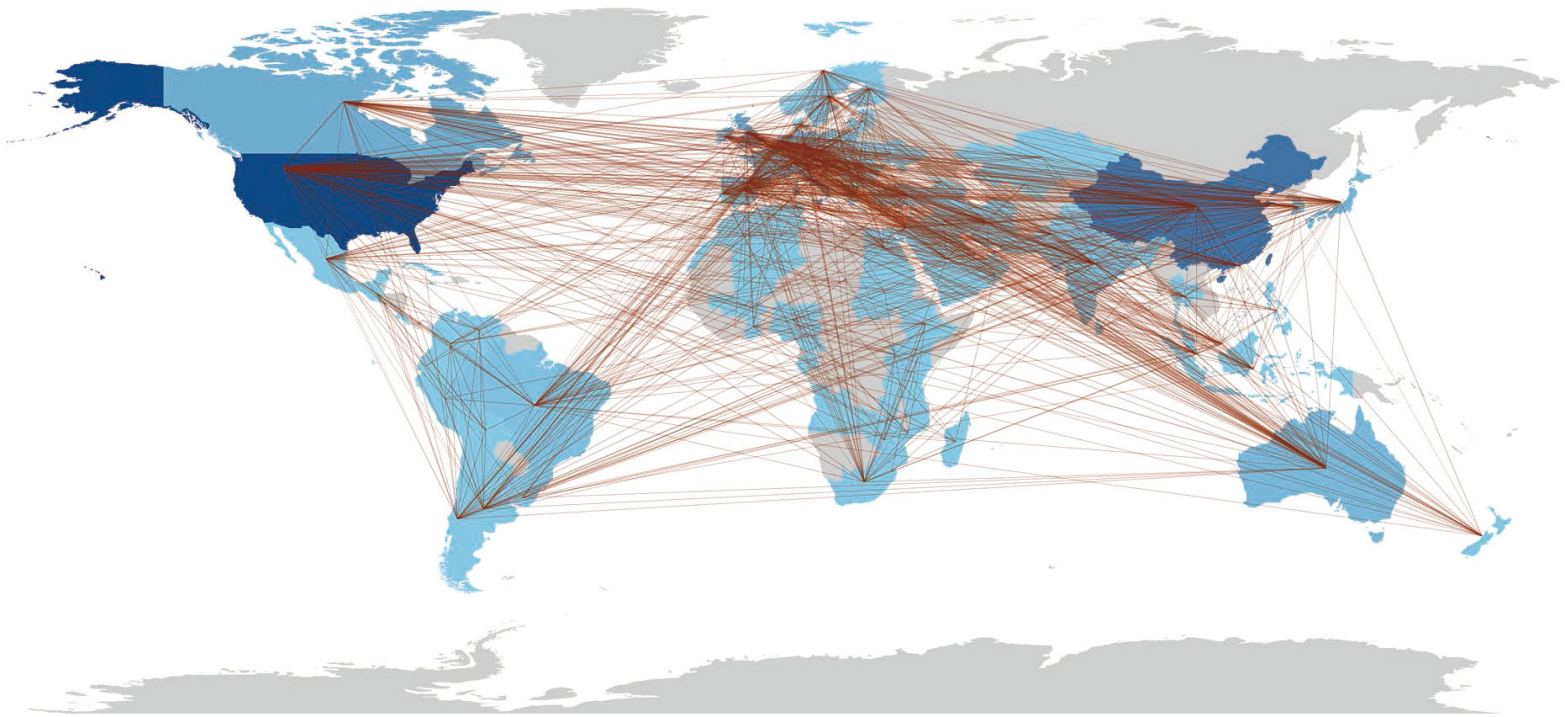
Trends in the publications on intestinal microbiota in IBD research involved 217 countries/regions over six continents. The interconnection among nations signifies collaborative efforts.

The geographical distribution of research productivity from 217 countries/regions on six continents was presented in **Figure 6**. In the map, the darker blue color represented countries/regions with the higher productivity of gut microflora and IBD articles. The intensity of the color is directly proportional to the quantity of publications. Among the most productive countries, the United States (US) contributed most to the research productivity (9196 publications), followed by China (5587), Italy (2305), the United Kingdom (1806), and France (1740). International collaboration of active countries/regions was also assessed and presented in a network visualization map (**Figures 6**, **S2**). The thickness of the link between any two countries/regions indicated the strength of collaboration, while the density of the threads assigned for that country/region indicated the extent of international collaboration. The network visualization map shows that the most vigorous collaboration was between the US and China. With the densest line, the US implied the greatest extent of international collaboration with 80 countries/regions due to the wide range of its publications. From the cluster analysis in the map, countries/regions such as the US and Canada were observed in a close cluster, while Germany, Sweden, and Denmark were found in another close cluster. The ranking of production and national collaborations between the corresponding author’s countries are shown in **Table S1**. Citations analysis for countries in **Table S2** showed that the US had been the most highly cited globally.

### Contribution of academic institutions

**Table 5** lists the top 20 prolific institutions publishing papers on gut flora and IBD. Harvard Medical School ranked first in productivity with 336 scientific publications, followed by the University of California (274) and University College Cork (208). The 20 most active institutions are primarily in North America, with 10 in the US and 4 in Canada. The other six institutions are widely distributed in Europe (Ireland, Belgium, Denmark), Oceania (Australia), and Asia (China). The analysis of 277 organizations with high citations of more than 1000 times is shown in **Figure S3**. The Division of Biology, California Institute of Technology, Pasadena, CA, United States received the highest citation number (7833 citations), while the FAS Center for Systems Biology, Harvard University, Cambridge, MA, in the United States (6599) and Laboratory of Microbiology, Wageningen University Wageningen in Netherlands (6272) were in the second and third place, respectively.

**Table 5:** Top 20 prolific institutions in publishing papers on gut flora and IBD.

SCR	Institution	Documents	Country	% N=10497
1^st^	Harvard Medical School	336	USA	3.2
2^nd^	University of California	274	USA	2.6
3^rd^	University College Cork	208	Ireland	2.0
4^th^	University of Alberta	168	Canada	1.6
5^th^	University of Toronto	163	Canada	1.5
6^th^	University of Calgary	162	Canada	1.5
7^th^	Baylor College of Medicine	139	USA	1.3
8^th^	University of North Carolina at Chapel Hill	138	USA	1.3
9^th^	Icahn School of Medicine at Mount Sinai	134	USA	1.3
10^th^	Mcmaster University	133	Canada	1.3
10^st^	University of Michigan	133	USA	1.3
12^st^	University of Pennsylvania	124	USA	1.2
13^rd^	Cornell University	121	USA	1.2
14^th^	University of Chicago	114	USA	1.1
15^th^	University of California San Diego	111	USA	1.1
15^th^	University of Copenhagen	111	Danmark	1.1
17^th^	Ghent University	109	Belgium	1.0
17^th^	Jiangnan University	109	China	1.0
19^th^	Zhejiang University	105	China	1.0
20^th^	University of New South Wales	103	Australia	1.0

### Analysis of research interests in terms of frequency

**Figure 7:**
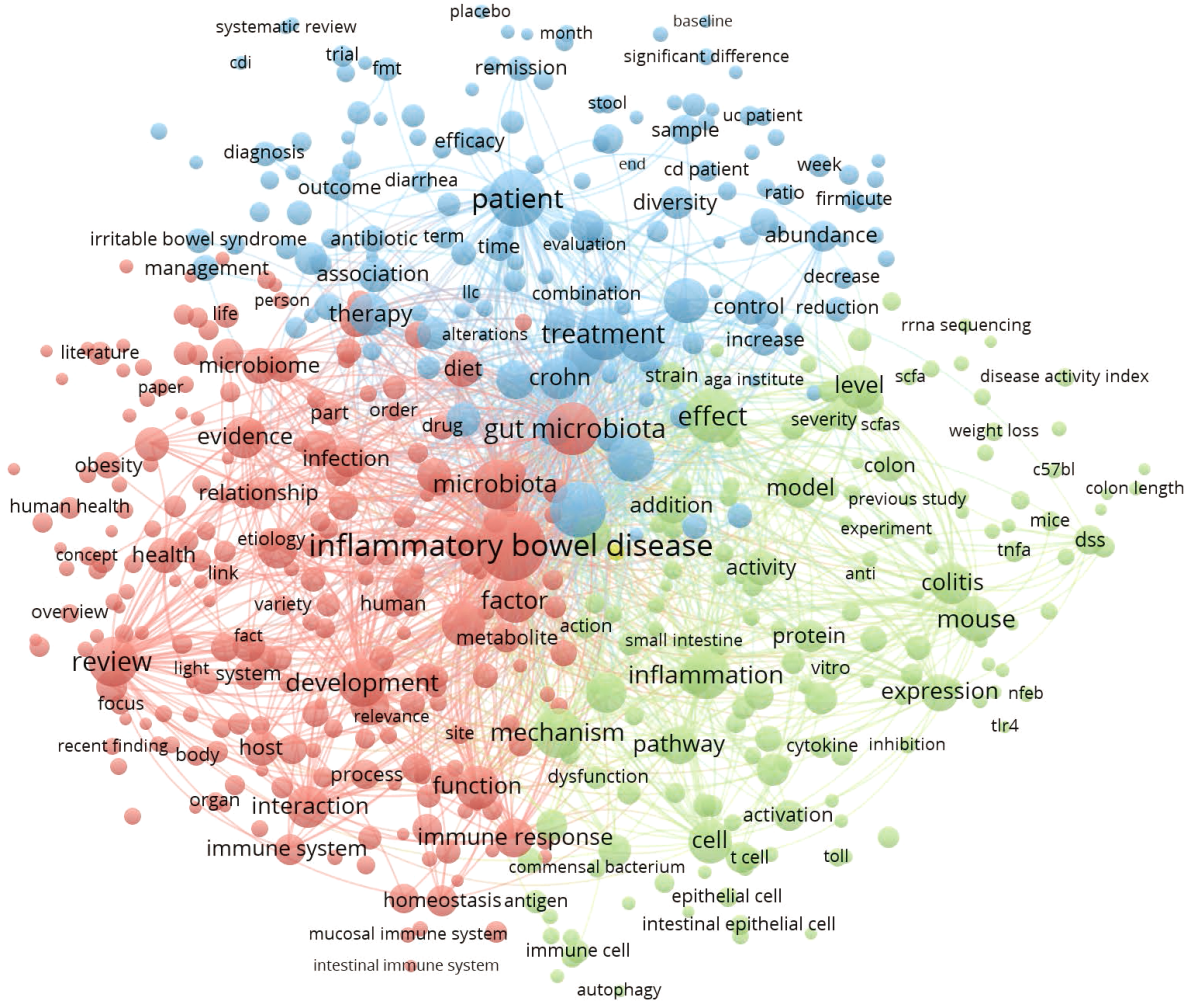
Density map of most frequently encountered terms extracted from the titles and abstracts of retrieved publications. A number of 525 terms met the threshold with a minimum number of occurrences of 100. The larger circle size or font size indicates higher occurrence.

This thematic analysis was performed on the terms that appeared in the information sources of retrieved publications from 2003-2022. The terms were mainly from the title, abstract, and keyword fields of academic literature, representing the authors’ main concepts and research interests for communication. A density visualization map is used to display which terms occur more often and how the terms interconnect (**Figure 7**). The larger the character fonts, the more frequently the terms are applied. A total of 525 terms that occurred more than 100 times were presented and divided into three groups with their interconnections. As a result, terms such as inflammatory bowel disease(IBD) were by far the most prevalent (8168 times), followed by patient (3202), gut microbiota (2651), microbiota (2346), and inflammation (2210).

## Discussion

### General research directions

IBD significantly impacts daily life and poses a significant risk for the development of gastrointestinal malignancies (3). Due to its clinical refractory nature, treating IBD has become a prominent topic in healthcare. However, the in-depth pathogenesis that accounts for IBD has been largely debated. The role of commensal microbiota in the onset and development of IBD has attracted increasing attention (6). Aberrant microbiota community structure and dysbiosis of the host’s microbiota may affect the gut’s immunological function and immune homeostasis (50). As metabolites of gut microbiota, short-chain fatty acid (SCFA) and bile acid have been shown to work on immune homeostasis through cell signaling receptors or epigenetic regulations (50, 51). The dysfunction of the intestinal barrier leading to pathological bacterial translocation also contributes to IBD (1).

The perspective of intestinal microbiota may shed new light on the investigation of IBD. Lifestyle and environmental factors could modulate the gut microbiota composition and bacterial diversity (52). Unhealthy dietary patterns result in microbiota dysbiosis, which becomes the facilitator of IBD (4). Gut microbiota-related approaches may have the potential to ameliorate IBD (7). Fecal microbiota transplantation has been shown to have beneficial effects, including alleviating colonic inflammation and promoting the restoration of intestinal homeostasis through multiple immune-mediated pathways (53). However, more in-depth investigations are needed to reach a thorough understanding of the interplay between intestinal microbiota and IBD.

Bibliometric analysis is a quantitative study of bibliographic information, including authors, institutions, publication types, source countries, funding and citation information, etc. (54). This approach can assess the academic performance of journals, authors, or countries and provide a comprehensive overview of a particular research domain. This study aimed to present a complete picture of intestinal microbiota and IBD research during the past two decades. The significance of gut microbiota in IBD-related research was investigated using a bibliometric method for the first time, and the scientific production and global trends of this field were also evaluated.

As revealed in the results, since 2003, Sartor R. Balfour, Colombel Jean Frederick, and Shanahan Ferguson have pioneered the field of gut microbiota and inflammation study, which has demonstrated sustained and exponential growth over the ensuing two decades. Especially in the last few years, the annual number of documents has been soaring since 2007, which coincides with the launch of the HMP and MetaHIT projects (55, 56). In recent years, gut microbiota research has been drawing increased attention to provide a new perspective on many complicated issues, it also accelerates the understanding of the origin and mechanism of IBD. The steady growth in the percentage of intestinal microbiota-related publications on IBD research indicates a promising future in this domain.

### Hotspots and Frontiers

Through the utilization of keyword cluster analysis and timeline view, the current research hotspots pertaining to IBD and intestinal flora can be primarily categorized into two aspects: the microscopic mechanisms underlying IBD and its clinical treatment, with a particular emphasis on exploring the inflammatory pathways mediated by intestinal flora. The intestinal barrier and mucosal immunity play crucial roles in connecting gut microbiota with IBD. The TNF-α, TLR4, and other signaling pathways, as well as the metabolic mechanisms of intestinal flora such as SCFA, have garnered increasing attention in recent years. In studies of gut microbes associated with IBD, proteobacteria, and parabacteroides were the most heavily investigated in the past two decades, which have been reported in 287 and 154 articles, respectively, revealing the research hotspots in this field. Human-centered clinical study is experiencing steady growth and is poised to become a prominent topic in this field.

### Existing limitations

This study solely focused on publications within the Scopus database and did not encompass other databases, such as PubMed and Web of Science, which may yield marginally distinct outcomes. Despite being the largest peer-reviewed abstracts and citation database globally, it is plausible that several papers on this topic may have been published in journals not incorporated in Scopus. The other limitation of our study, which is inherent to any bibliometric approach, is that we did not examine individual article records beyond the random sample used for verifying index accuracy. Instead, we relied on MEDLINE indexes for classification purposes. Nevertheless, the manually verified 5% sample had a high level of accuracy; thus, we can confidently assert that the results obtained through bibliometric analysis are valid.

## Conclusions

This extensive bibliometric study provides researchers with a global overview of academic trends, geographical distribution, and collaboration patterns in the field of gut microbiota and IBD research over the past 20 years. The comprehensive analysis and structured data presented in this study benefit scientists in screening academic interests and informing policymakers in developing policies related to this topic. Furthermore, the results reveal the significant role of gut microbiota in IBD and lay the groundwork for further explorations in the future.

## Data availability statement

The original contributions presented in the study are included in the article/**Supplementary Material**, further inquiries can be directed to the corresponding author/s.

## Author contributions

AZ: Writing – original draft, Writing – review & editing. FW: Writing – review & editing. DL: Writing – review & editing. C-ZW: Writing – review & editing. HY: Writing – original draft, Writing – review & editing. J-YW: Writing – review & editing, Writing – original draft. C-SY: Writing – review & editing.
