# Supplementary material for: Emerging insights into inflammatory bowel disease from the intestinal microbiota perspective: a bibliometric analysis
Source: Front Immunol. 2023 Oct 26;14:1264705. doi: 10.3389/fimmu.2023.1264705 (PMC10639163; doi:10.3389/fimmu.2023.1264705)
Supplement: Supplementary file 1 [file DataSheet_1.docx]

**Supplementary materials**

Emerging Insights into Inflammatory Bowel Disease from the Intestinal Microbiota Perspective: A Bibliometric Analysis

Anqi Zhang^1,2^, Delong Li^1,2^, Chong-Zhi Wang^3,4^, Haiqiang Yao^1,2*^,

Jin-Yi Wan^1,2,*^, Chun-Su Yuan^3,4^

*^1^ School of Traditional Chinese Medicine, Beijing University of Chinese Medicine, Beijing 100029, China*

*^2^ National Institute of TCM Constitution and Preventive Medicine, Beijing University of Chinese Medicine, Beijing 100029, China*

*^3^ Tang Center for Herbal Medicine Research, The University of Chicago, Chicago, IL 60637, USA*

*^4^ Department of Anesthesia and Critical Care, The University of Chicago, Chicago, IL 60637, USA*

* Correspondence to: Jin-Yi Wan, wanjinyi1128@163.com (ORCID 0000-0001-8830-2035), Haiqiang Yao, haiqiangyao@outlook.com (ORCID: 0000-0001-9716-0171).

This supplementary file contains: Fig. S1-S4, Table S1-S2.

**
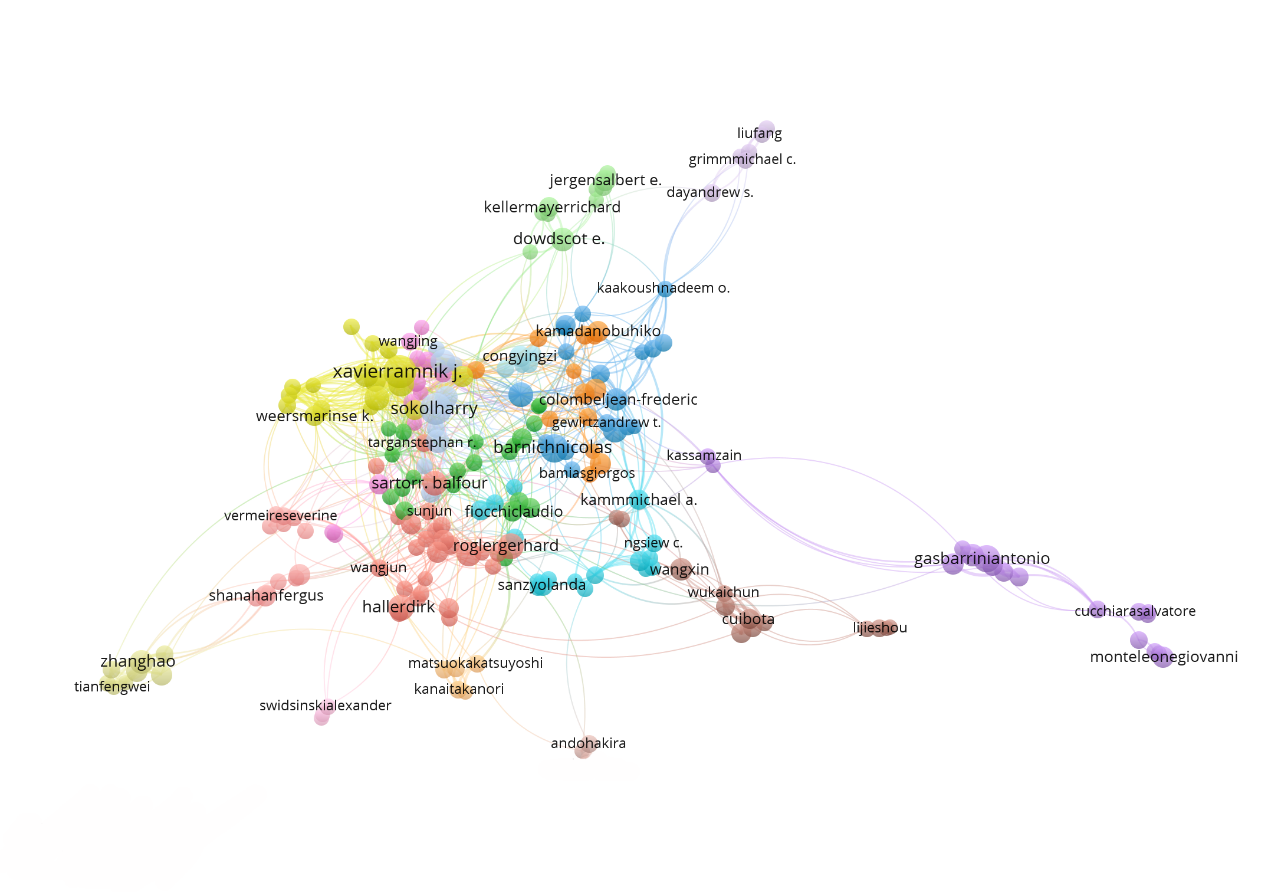
**

**Fig. S1.** Citation analysis of 234 authors with at least ten publications. Thicker lines indicate stronger collaborations. Authors represented with larger circle size or font size have relatively more citations


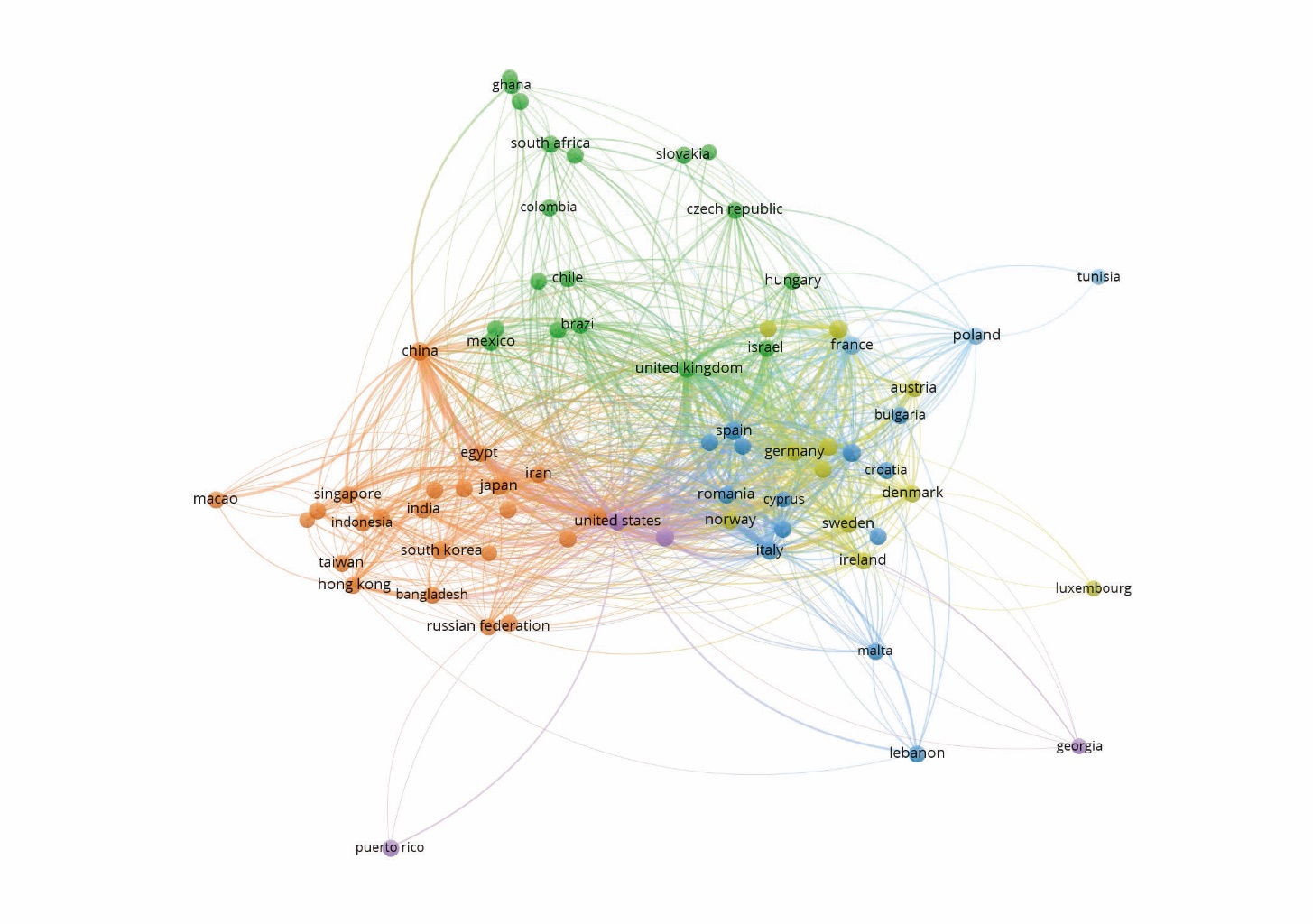


**Fig. S2.** Cooperation between countries. The density of the lines reflects the frequency of cooperation.


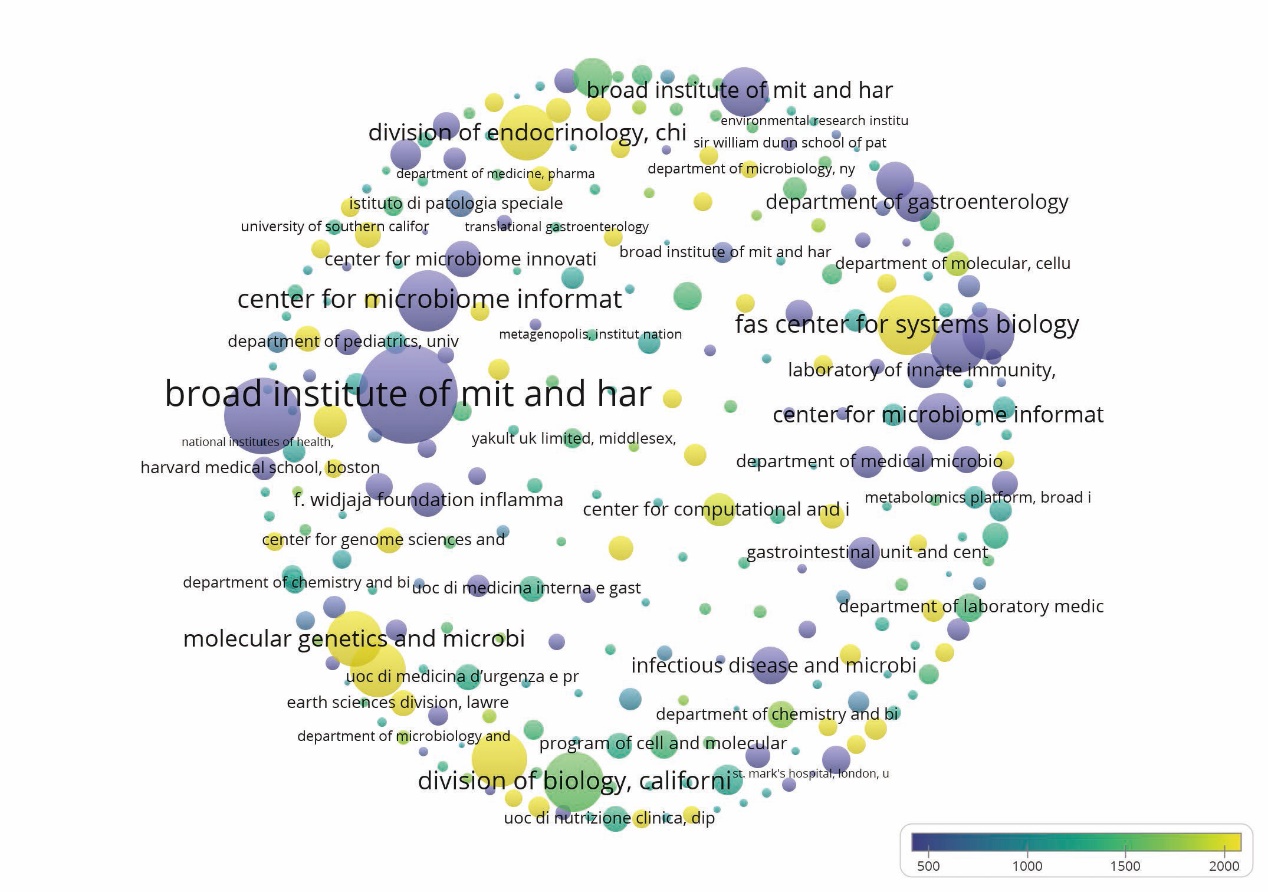


**Fig. S3.** Density map of 277 organizations with high citations of more than 1000 times. The dot size is directly proportional to the number of articles published. The degree of yellowness corresponds to the number of citations received, while shades of blue indicate fewer citations.


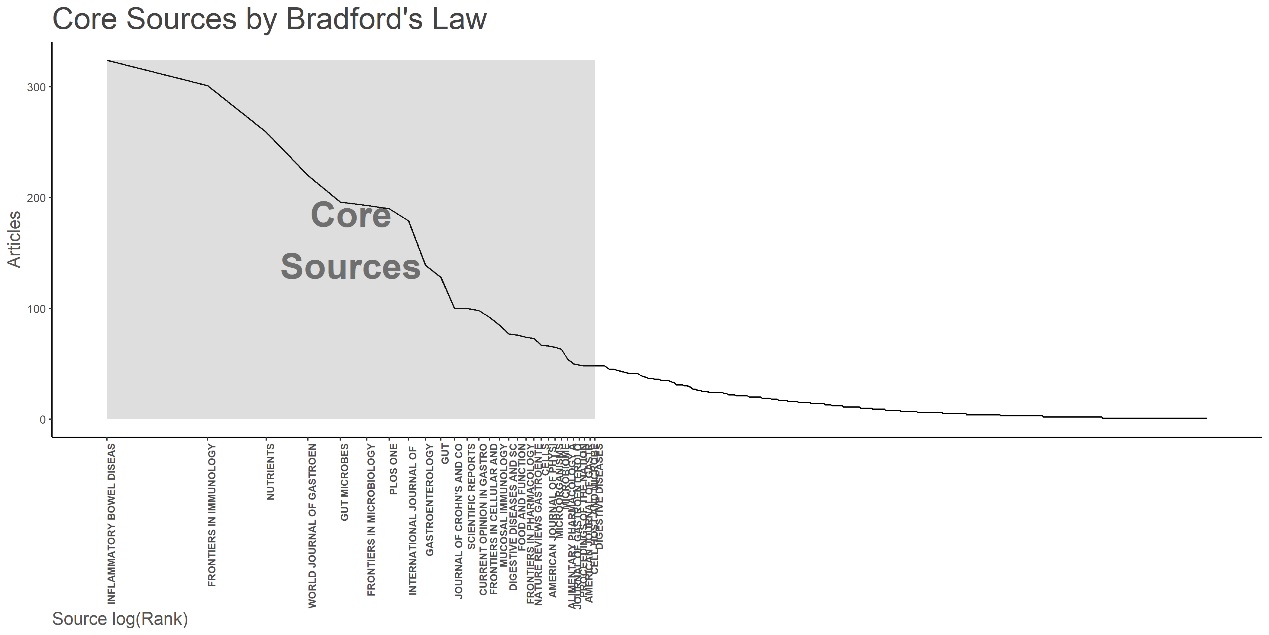


**Fig. S4.** Core sources by Bradford’s law. The x-coordinate represents the periodicals within the central domain, while the y-coordinate indicates the number of articles.

**Table S1.** Top 20 countries ranked by collaborations based on corresponding authors.

| **Rank** | **Country/Region** | **Articles** | **SCP** | **MCP** | **Freq** | **MCP Ratio** |
| --- | --- | --- | --- | --- | --- | --- |
| 1^st^ | USA | 2197 | 1681 | 516 | 0.212 | 0.235 |
| 2^nd^ | China | 1745 | 1425 | 320 | 0.168 | 0.183 |
| 3^rd^ | Italy | 641 | 480 | 161 | 0.062 | 0.251 |
| 4^th^ | Germany | 393 | 244 | 149 | 0.038 | 0.379 |
| 5^th^ | United Kingdom | 462 | 319 | 143 | 0.045 | 0.31 |
| 6^th^ | Canada | 407 | 274 | 133 | 0.039 | 0.327 |
| 7^th^ | France | 336 | 209 | 127 | 0.032 | 0.378 |
| 8^th^ | Australia | 249 | 166 | 83 | 0.024 | 0.333 |
| 9^th^ | Netherlands | 180 | 99 | 81 | 0.017 | 0.45 |
| 10^th^ | Switzerland | 128 | 69 | 59 | 0.012 | 0.461 |
| 10^st^ | Spain | 228 | 177 | 51 | 0.022 | 0.224 |
| 12^st^ | Ireland | 131 | 86 | 45 | 0.013 | 0.344 |
| 13^rd^ | Sweden | 106 | 64 | 42 | 0.01 | 0.396 |
| 14^th^ | Japan | 303 | 264 | 39 | 0.029 | 0.129 |
| 15^th^ | Belgium | 116 | 77 | 39 | 0.011 | 0.336 |
| 15^th^ | Iran | 101 | 62 | 39 | 0.01 | 0.386 |
| 17^th^ | Denmark | 102 | 67 | 35 | 0.01 | 0.343 |
| 17^th^ | Israel | 100 | 66 | 34 | 0.01 | 0.34 |
| 19^th^ | India | 200 | 167 | 33 | 0.019 | 0.165 |
| 20^th^ | Brazil | 109 | 84 | 25 | 0.011 | 0.229 |

Abbreviations: SCP, single country publication; MCP, multi-country publication. Country denotes the affiliation of the corresponding author.; Articles denote the number of publications per country based on the corresponding author's affiliation.

**Table S2.** Top 20 most cited countries on gut flora and IBD.

| Rank | **Country/Region** | **Total Cited** | **Average Article Citations** |
| --- | --- | --- | --- |
| 1^st^ | USA | 201559 | 91.70 |
| 2^nd^ | China | 46798 | 26.80 |
| 3^rd^ | United Kingdom | 40009 | 86.60 |
| 4^th^ | France | 32010 | 95.30 |
| 5^th^ | Germany | 31142 | 79.20 |
| 6^th^ | Canada | 29032 | 71.30 |
| 7^th^ | Italy | 27156 | 42.40 |
| 8^th^ | Spain | 16637 | 73.00 |
| 9^th^ | Ireland | 14055 | 107.30 |
| 10^th^ | Australia | 14029 | 56.30 |
| 10^st^ | Belgium | 12562 | 108.30 |
| 12^st^ | Japan | 12074 | 39.80 |
| 13^rd^ | Netherlands | 10847 | 60.30 |
| 14^th^ | Switzerland | 7107 | 55.50 |
| 15^th^ | Korea | 6572 | 35.30 |
| 15^th^ | Sweden | 6513 | 61.40 |
| 17^th^ | Israel | 6082 | 60.80 |
| 17^th^ | India | 5598 | 28.00 |
| 19^th^ | Denmark | 4937 | 48.40 |
| 20^th^ | Poland | 3561 | 22.00 |
